# Therapeutic potential of omega-3 fatty acids supplementation in a mouse model of dry macular degeneration

**DOI:** 10.1136/bmjophth-2016-000056

**Published:** 2017-06-19

**Authors:** Ekatherine Prokopiou, Panagiotis Kolovos, Maria Kalogerou, Anastasia Neokleous, Gregory Papagregoriou, Constantinos Deltas, Stavros Malas, Tassos Georgiou

**Affiliations:** 1 Ophthalmos Research and Educational Institute, Nicosia, Cyprus; 2 Department of Biological Sciences, Molecular Medicine Research Centre and Laboratory of Molecular and Medical Genetics, University of Cyprus, Nicosia, Cyprus; 3 Developmental and Functional Genetics Group, The Cyprus Institute of Neurology and Genetics, Nicosia, Cyprus

**Keywords:** omega-3 fatty acids, inflammation, macular degeneration, retina, photoreceptors.

## Abstract

**Purpose:**

To evaluate the therapeutic effects of omega-3 (ω-3) and omega-6 (ω-6) fatty acids in the CCL2^−/−^ model of dry age-related macular degeneration (AMD). The blood level of eicosapentaenoic acid (EPA) and arachidonic acid (AA) served to adjust the treatment dosage (AA/EPA=1–1.5).

**Methods:**

Nine-month-old animals were allocated to different groups: (A) C57BL/6 untreated , (B) CCL2^−/−^ untreated, (C) CCL2^−/−^ treated with ω-3+ω-6, and (D) CCL2^−/−^ treated with ω-3. Treatment was daily administered by gavage for 3 months. Fatty acids analysis was performed and retinas were histologically examined. Three-month-old wild type mice were used for comparison purposes. Real-time PCR and Western blot were performed for retinal inflammatory mediators.

**Results:**

Increased EPA and decreased AA levels were observed in both blood and retinas in the treatment groups. The outer nuclear layer thickness was increased in groups C (45.0±3.9 µm) and D (62.8±4.9 µm), compared with groups B (65.6±3.0 µm) and A (71.1±4.2 µm), and in younger mice, it was 98.0±3.9 µm. A decrease in NF-κB expression was noted in the treatment groups. Interleukin (IL) 18 protein levels demonstrated a significant reduction in the ω-3-treated group only.

**Conclusion:**

Supplementation with ω-3+ω-6 or ω-3 alone (AA/EPA=1–1.5) suggests a protective mechanism in the CCL2^−/−^ animal model of dry AMD, with a more beneficial effect when ω-3 are used alone. Our findings indicated that inflammation is not the only determining factor; perhaps a regenerative process might be involved following administration of ω-3 fatty acids.

**Key messages**

**What is already known about the subject?**

- Age-related macular degeneration (AMD) is the leading cause of visual impairment and blindness in the elderly. Currently, there are no guidelines for the first-line treatment of dry AMD; however, several anti-oxidants, vitamins and zinc may reduce its progression.
- Several studies have demonstrated that omega-3 (ω-3) fatty acids may have a protective role in inflammatory-associated, ischaemia-associated, light-associated, oxygen-associated and age-associated pathology of the vascular and neural retina. Recently, Epitropoulos *et al* assessed the effect of oral ω-3 fatty acids (1680 mg eicosapentaenoic acid (EPA) and 560 mg docosahexaenoic acid (DHA), for 12 weeks) in a multi-centre, placebo-controlled, double-masked study in patients with dry eyes. The authors reported a significant improvement in several parameters, including tear osmolarity and tear break-up time. In addition, Georgiou and Prokopiou reported the results from an observational study, where patients with dry AMD were supplemented with EPA and DHA for up to 6 months (5–7.5 g/day EPA and DHA, arachidonic acid (AA)/EPA<2) and demonstrated significant improvement in vision (≥15 letters gain).

**What are the new findings?**

- In this study, we investigated the effects of ω-3 fatty acids alone or in combination with omega-6 (ω-6) in the CCL2^−/−^ model of AMD while monitoring the blood levels of EPA and AA (AA/EPA=1–1.5). Supplementation with ω-3+ω-6 or ω-3 alone (when AA/EPA=1–1.5) suggests a protective mechanism in the CCL2^−/−^ animal model of dry AMD, with a more beneficial effect when ω-3 are used alone. Retinal photoreceptors’ layer demonstrated a highly significant increase in the ω-3-treated group compared with the untreated. Inflammatory mediators showed decreased levels at the mRNA and protein level in the treatment groups. Our findings indicated that inflammation is not the only determining factor; perhaps a regenerative process might be involved following administration of ω-3 fatty acids.

**How might these results change the focus of research or clinical practice?**

- These findings are of great importance since supplementation with ω-3 fatty acids at the right dosage (blood AA/EPA=1–1.5) could be beneficial in patients with AMD and perhaps other ocular pathologies which involve photoreceptor damage. The benefits will have a positive impact on the quality of life of patients suffering from such conditions.

## Introduction

Age-related macular degeneration (AMD) is the leading cause of visual impairment and blindness in the elderly; an estimated 200 million people suffer from AMD worldwide. By the year 2040, the number of these individuals is estimated to increase by 50%.^1 2^ Currently, there is no definitive pathogenesis model for AMD, but several different aetiologies have been proposed. Ageing is one of the most common contributing factors in AMD due to the accumulation of oxidised lipoproteins and free radicals in the retina and choroid. This accumulation in turn results in oxidative stress and a decrease in the number of retinal pigment epithelium (RPE) cells and photoreceptors.^3 4^ Genetic predisposition, as with multiple pathologies, plays a role in the development of AMD. Although there are several genetic associations, the most studied are certain polymorphic loci, in particular, those related to inflammatory genes such as complement factor H and certain complement components (eg, C3 and C2).^5 6^ Environmental factors, including smoking, sunlight exposure, high-fat diet, obesity and diabetes, are all associated with the development and progression of AMD.^7^

Furthermore, a tissue-adaptive response, recently described as para-inflammation, in which the innate immune system mounts a low-grade inflammatory response to restore tissue homeostasis, has been implicated in the pathogenesis of AMD.^8 9^ Inflammatory-related proteins, including C-reactive protein,^10–13^ interleukin-6 (IL-6)^12 14^ and tumour necrosis factor-α (TNF-α),^12 15^ have been shown to be associated with AMD; however, the results from different groups are inconsistent. The fact that systemic inflammatory markers are not strongly related with AMD suggests that local low-grade inflammation is more likely to be involved in its pathogenesis.

Over the past few decades, there has been an increasing interest in the role of omega-3 (ω-3) polyunsaturated fatty acids (PUFAs) in inflammation. Evidence from preclinical and clinical studies has proven the effectiveness of ω-3 PUFAs against heart disease, cancer, diabetes and neurological and autoimmune diseases.^16^ To date, several studies have focused on the therapeutic role of ω-3 PUFAs, which are considered anti-inflammatory molecules. The resolution of inflammation is an active process primarily driven by a new family of mediators, termed resolvins, derived from the ω-3 PUFAs eicosapentaenoic acid (EPA, C20:5 ω-3) and docosahexaenoic acid (DHA, C22:6 ω-3).^17^

Among the major mediators of the inflammatory response is the generation of pro-inflammatory eicosanoids generated from the omega-6 (ω-6) PUFA arachidonic acid (AA, C20:4 ω-6). These mediators include pro-inflammatory prostaglandins (eg, PGE2) and leukotrienes (eg, LTB4), which can act as mediators for leucocyte chemotaxis and inflammatory cytokine production. The balance between the pro-inflammatory and anti-inflammatory molecules plays a key role in disease progression and the resolution of an inflammatory response.

Several studies have demonstrated that ω-3 PUFAs may have a protective role in inflammatory-associated, ischaemia-associated, light-associated, oxygen-associated and age-associated pathology of the vascular and neural retinas.^18^ Administration of PUFAs was previously found to have a promising effect in several animal models of macular degeneration.^19–23^

Currently, there are no guidelines for the first-line treatment of dry AMD; however, several anti-oxidants, vitamins and zinc may reduce its progression according to the Age-related Eye Disease Study (AREDS).^24^ Following the AREDS, an additional study was performed, the AREDS2, which was a multi-centre 5-year randomised trial designed to examine the effects of oral supplementation of macular xanthophylls (10 mg lutein and 2 mg zeaxanthin) and/or ω-3 PUFAs (EPA 650 mg and DHA 350 mg) on the progression to advanced AMD. Overall, there was no additional benefit from adding the ω-3 PUFAs or a mixture of lutein and zeaxanthin to the formulation. Although the addition of ω-3 to the AREDS formulation was not shown to be beneficial, it is believed that higher doses of EPA and DHA may have a desirable effect.^25^

Therefore, further studies were conducted or are still ongoing to investigate the possible mechanisms of action of PUFAs and to examine any positive effects on disease progression. Recently, Epitropoulos *et al* assessed the effect of oral ω-3 PUFAs (1680 mg EPA and 560 mg DHA, for 12 weeks) in a multi-centre, placebo-controlled, double-masked study in patients with dry eyes. The authors reported a significant improvement in several parameters, including tear osmolarity and tear break-up time.^26^ In addition, Georgiou and Prokopiou reported the results from an observational study, where patients with dry AMD were supplemented with EPA and DHA for up to 6 months (5–7.5 g/day EPA and DHA, AA/EPA <2) and demonstrated significant improvement in vision (≥15 letters gain).^27^

The importance of particular fatty acids, such as the blood levels of EPA and AA, has recently been emphasised. In particular, the Japan EPA Lipid Intervention Study established the clinical efficacy of EPA for cardiovascular disease (CVD), in which higher levels of EPA but not DHA were found to be associated with a lower incidence of major coronary events. The risk of coronary events was significantly reduced when the ratio of EPA to AA (EPA/AA) was >0.75.^28^ In addition, the ratio of PGI3 and PGI2 (which both reduce cardiac ischaemic injury and arteriosclerosis and promote angiogenesis) to thromboxane A2 was determined to have a linear relationship with the EPA/AA ratio. Thus, the effects of EPA in reducing the risk of CVD could be mediated by the biological action of PGI3 in addition to the hypo-triglyceridemic action of EPA.^29^ A number of studies have confirmed the association of EPA/AA both in coronary diseases and in diabetes and hyperlipidaemia.^30 31^

Lower levels of particular PUFAs in either circulating blood or the retina are associated with some retinopathies. In another study, eyes from AMD donors exhibited significantly decreased levels of very long-chain PUFAs and high ω-6/ω-3 ratios.^32^ Therefore, examining systemic biomarkers (including the levels of AA and EPA) when undertaking therapeutic trials could be a good indication of disease progression and treatment success. However, further studies are required to better establish the relationship between the level of certain fatty acids and the progression of ocular pathologies.

In this study, we investigated the effects of ω-3 PUFAs in the CCL2^−/−^ model of AMD while monitoring the blood levels of EPA and AA (AA/EPA=1–1.5). To date, no ideal animal model has been established that fully recapitulates the human features of AMD; however, the CCL2^−/−^ model shares certain common characteristics. There have been conflicting reports between different studies, some suggesting that most of the previously described hallmark features of AMD in the CCL2^−/−^ model can be explained by normal ageing.^33^ Others indicated that this model develops drusen and other features of AMD, such as the accumulation of lipofuscin in RPE cells, progressive outer retinal degeneration and geographic/RPE atrophy.^34^

## Materials and methods

### Animals

Age-matched (9 months old) CCL2^−/−^ (B6.129S4-*Ccl2^tm1Rol^*/J, Jacksons Laboratory, USA) and C57BL/6 mice (3 months old and 9 months old, considered wild type, CY/EXP/101 breeding colony) of both sexes were used in this study and kept under standard housing conditions with a 12-hour dark/light cycle and with food and water provided *ad libitum*. All experiments were performed in accordance with the animal care protocols approved by the Cyprus Government’s Chief Veterinary Officer according to EU guidelines.

### Study design

The animals were randomly allocated to four different groups (n=22–24 in each group): (A) wild type untreated C57BL/6, (B) age-matched untreated CCL2^−/−^, (C) age-matched CCL2^−/−^ treated with ω-3+ω-6 (100 mg EPA+50 mg DHA +30 mg γ-linolenic acid ((GLA), KD Pharma, Germany and Ophthalmos Omega 3, Cyprus) and (D) age-matched CCL2^−/−^ treated with ω-3 only (130 mg EPA, KD Pharma, Germany and Ophthalmos Omega 3, Cyprus). The animals in the treatment groups were administered fish oil daily by gavage for 3 months. The oral dosing total volumes did not exceed 10 mL/kg. The general health and appearance of the animals were assessed daily, and body weight measurements were recorded weekly. The mice were euthanised at the end of the study by cervical dislocation.

### Fatty acid analysis

To determine the AA/EPA ratio, following long-term treatment, blood samples (n=20–24) were collected on D0, D30 and D90, prior to treatment, processed for fatty acids separation and analysed using gas chromatography (GC). In addition, after completion of the study (D90), retinas (n=4) were collected from each group for the same purpose. Blood samples were collected on a Whatman filter paper and stored at −20°C. In each sample, NaOH/MeOH was added and heated for 1 min at 85°C, and then 14% BF3/MeOH was added and heated for 7 min at 85°C. Once the samples reached room temperature, 0.3 g of NaCl was added to ensure complete migration of the total fatty acid methyl ester fraction to the organic phase, followed by 1 mL of n-hexane. When the samples were separated into the organic and aqueous phases, the organic phase was collected and this was repeated three times. To remove any impurities, 0.9 g Na_2_SO_4_ was added to the samples and then centrifuged at 3300 r.p.m. for 5 min. Supernatants were collected and dried using an analytical evaporator at ~45°C under a nitrogen stream. Once dried, the samples were redissolved in n-hexane and analysed using a GC flame ionisation detector (FID).

### Gas chromatography flame ionisation detector

An Agilent GC-6890 system was equipped with an FID. An H_2_ flow rate of 35 mL/min and an air flow rate of 350 mL/min were used. The flow rate of carrier gas (He) was set at 2.5 mL/min. The temperatures of the injection port and detector were set at 280°C and 300°C, respectively. The oven temperature was programmed to initiate at 160°C for 3 min, and then the temperature was raised to 200°C at a rate of 20°C/min, held there for 4 min and finally increased to 250°C at a rate of 5°C/min and held there for 23 min. The injection volume was 1 µL in the split-less injection mode. A capillary column (DP-23 fused-silica capillary, 30 m×0.25 mm I.D. x 0.25 µm film thickness; Supelco, Bellefonte, Pennsylvania, USA) was employed.

### Immunofluorescence staining

To assess the effect of treatment on the retinal photoreceptors (ie, the outer nuclear layer (ONL)), eyes from each group (n=5) were used. An additional group of younger (3-month-old) mice served as an additional control for comparison purposes. Eyes were enucleated at the end of the study (D90) and fixed with 2% paraformaldehyde for 2 hours, then transferred to a 30% sucrose solution in PBS for 24 hours at 4°C and embedded in optimum cutting temperature media (Sakura Finetek, Torrance, California, USA). Whole eyes were cross-sectioned and sections were obtained throughout the eye, including inferior pole, the optic nerve and superior pole. A total of 20 sections were taken, 28 µm apart, throughout the whole eye, including peripheral and central retinal areas. Briefly, 14-µm-thick cryosections were blocked with 10% donkey serum in 1% Triton X-100/PBS for 1 hour. Sections were then incubated with rabbit polyclonal anti-cone arrestin (AB15282, Chemicon, Temecula, California, USA; dilution 1:10 000) and mouse monoclonal anti-rhodopsin (clone 4D2, Chemicon; dilution 1:500) overnight at 4°C. After washing, sections were incubated for 2 hours with Alexa Fluor 594 donkey anti-rabbit IgG (1:200, Invitrogen) or Alexa Fluor 488 donkey anti-mouse IgG (1:200, Invitrogen). TO-PRO-3 iodide (T3605, Thermo Fisher Scientific, Massachusetts, USA) was used for nuclear staining. Samples were cover-slipped with Dako fluorescence mounting medium (Dako, Denmark) and examined by Leica TCS SP5 confocal microscopy (Leica, Germany). Measurements of the ONL were made at different fields around the entire retinal section (centre, middle and periphery). ImageJ software (NIH, http://imagej.nih.gov/ij) was used to calculate the ONL thickness, and the mean ONL thickness of the entire retina was compared among the different groups. The density of the photoreceptors was estimated as the number of photoreceptors nuclei over the retinal area, using ImageJ software.

### Quantitative reverse transcription PCR

On D90, retinas (n=4–5) from each group were excised, and tissue RNA was extracted using a NucleoSpin RNA/Protein kit (Macherey-Nagel, USA). The quantity and quality of RNA were determined spectrophotometrically (Nanodrop Technologies, Montchanin, DE). The same amount of total RNA was used for reverse transcription using a ProtoScript(R) II First-Strand cDNA Synthesis Kit according to the standard protocol of the supplier (New England Biolabs, UK). The quantitative PCR amplifications were performed on a ViiA 7 Real-Time PCR System (Applied Biosystems, USA) using Fast SYBR Green Master Mix (Thermo Fisher Scientific, USA) in a reaction volume of 20 µL. Relative quantification analysis was carried out on ViiA seven software. The primer sets for the genes whose differential expression was analysed by Quantitative reverse transcription PCR (qRT-PCR) are shown in table 1. Expression levels of each target gene were normalised to those of β-actin mRNA. Data are reported as the mean relative expression levels ± SE of the mean (SEM).

**Table 1:** Primer sequences of genes confirmed by quantitative reverse transcription PCR

Gene	Forward	Reverse
**TLR3**	TGCAGAAGATTCAAGGTACATCA	CAAACAGAGTGCATGGTTTAGTT
**NF-κB**	ACGAGGCAGCACATAGATGAΑ	AGGATGTCTCCACACCACTGTC
**IL-18**	GCCGACTTCACTGTACAACC	TCTGGTCTGGGGTTCACTGG
** β-Actin**	GCCTTCCTTCTTGGGTATGG	CGGATGTCAACGTCACACTT

### Western blot analysis

Retinal protein extracts were obtained from dissected retinas in each group (n=4–5) using a NucleoSpin RNA/Protein kit (Macherey-Nagel, USA). Twenty micrograms of total protein was detected using Qubit protein assay kit (Thermo Fisher Scientific, Massachusetts, USA) and β-actin was used as a loading control. The antibodies used against inflammatory mediators were as follows: 1) rabbit anti-IL-18 polyclonal antibody (5180 R-100, Biovision; dilution 1:1000), 2) goat anti-IL-17 polyclonal antibody (E-19, sc-6077, Santa Cruz Biotechnology, USA, dilution 1:200) and 3) goat anti-TNF-α polyclonal antibody (L-19, sc-1351, Santa Cruz Biotechnology, USA, dilution 1:200) followed by peroxidase-labelled secondary antibodies — either goat anti-rabbit IgG-HRP antibody (7074, Cell Signalling Technology; dilution 1:2,000) or donkey anti-goat IgG-HRP antibody (sc-2020, Santa Cruz Biotechnology, USA, dilution 1:2000). Proteins were detected using the Enhanced ChemiLuminescence Plus Blotting Detection system (Amersham Biosciences, Buckinghamshire, UK) and were visualised on a Chemidoc XRS+ imager (BioRad, USA). All immunoblots were re-probed with anti-β-actin antibody (D6A8, Cell Signalling Technology; dilution 1:1000) to confirm that equal amounts of protein were loaded on the membrane. The band density was defined using ImageJ Software. The results were expressed as the percentage density ratio of the protein of interest over the loading control (β-actin). All results were normalised against the untreated samples.

### Statistical analysis

All statistical analyses were performed using GraphPad Prism (V.5) statistical software. Data are expressed as the mean±SEM; the two-tailed Student’s t-test or one-way ANOVA with post hoc test: Tukey’s test was used to evaluate the differences among treated groups. Statistical significance was set at p<0.05.

## Results

### General observations

Animals’ general appearance was monitored daily and nothing abnormal was noted. Body weight measurements were recorded weekly, where no significant difference was found over time in any of the groups.

### Blood fatty acid analysis

**Figure 1:**
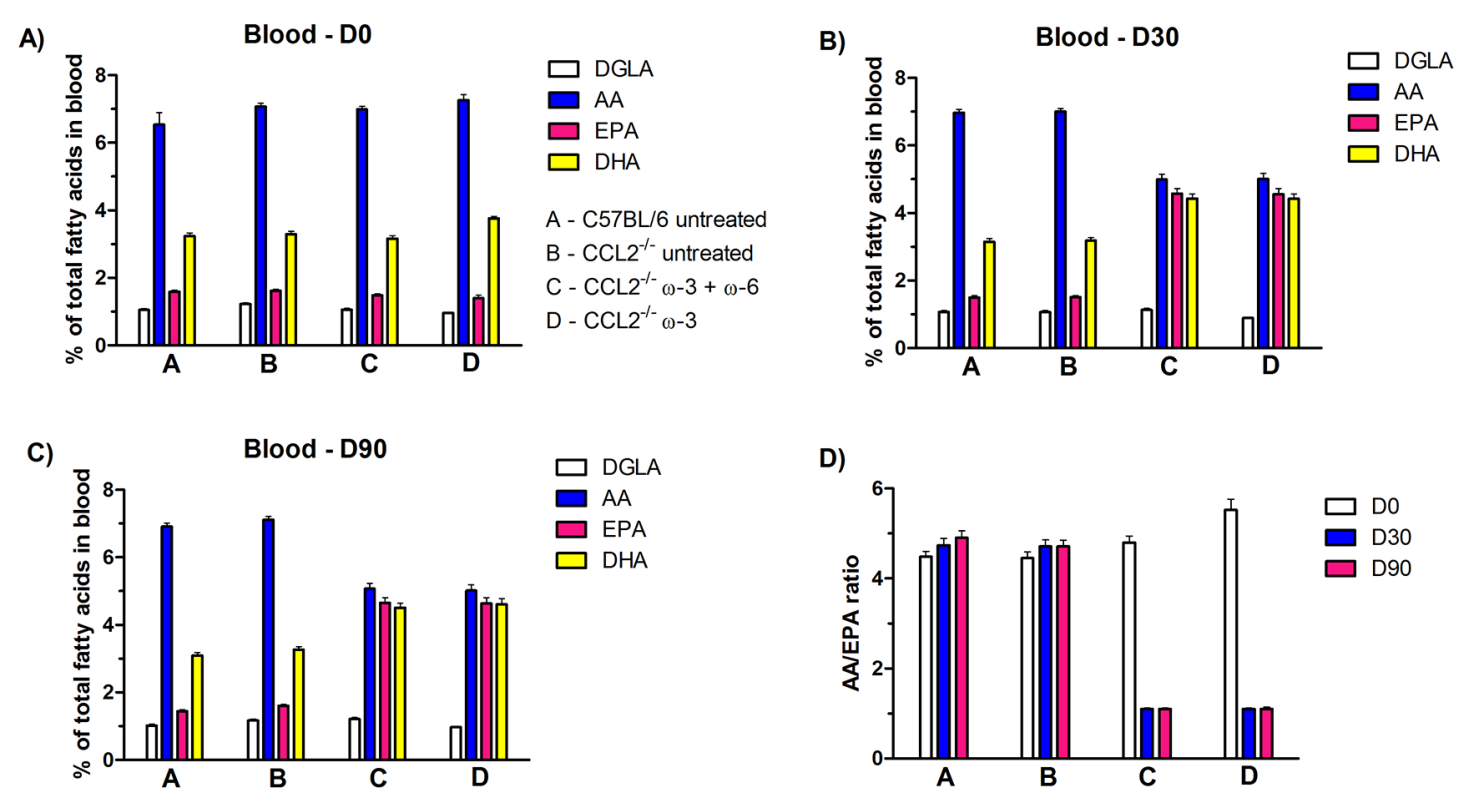
Fatty acid analysis from blood samples collected on (A) D0, (B) D30 and (C) D90 from C57BL/6 untreated group, CCL2^−/−^ untreated group, CCL2^−/−^ ω-3+ω-6-treated group and CCL2^−/−^ ω-3-treated group. (D) The AA/EPA ratio is shown in each group for D0, D30 and D90. The proportion of DGLA, AA, EPA and DHA is expressed as the mean±SEM (n=22–24).

For the duration of the study, blood fatty acids were monitored, as shown in figure 1 and table 2, in terms of percentage of total fatty acids. Samples were taken on D0, D30 and D90 prior to treatment. The levels of GLA could not be accurately detected; for that reason, dihomo-GLA (DGLA, a metabolite of GLA) was used for the purposes of this study. The percentage of the ω-6 fatty acids, DGLA and AA, as well as the percentage of the ω-3 fatty acids EPA and DHA in blood, was determined using a gas chromatographic technique (figure 1). The AA/EPA ratio was also calculated in each group, as shown in figure 1D. On D0, there was significantly lower levels of DGLA (p<0.01) and EPA (p<0.05) in the C57BL/6 mice compared with those in the CCL2^−/−^ untreated mice. The general trend observed was that, on D30, the level of EPA significantly increased (p<0.0001) in both treatment groups, whereas the level of AA was reduced (p<0.0001) compared with CCL2^−/−^ untreated and wild type groups. In particular, on D30, the blood AA/EPA ratio was decreased (p<0.0001) in both treatment groups (1.10±0.02 for both) compared with the CCL2^−/−^ untreated (4.71±0.15) and wild type (4.74±0.15) groups. Higher DHA levels (p<0.0001) were detected on D30 in the ω-3+ω-6-treated group (4.43±0.14) and in the ω-3-treated group (4.42±0.15) than in the CCL2^−/−^ untreated (3.18±0.09) and wild type (3.15±0.09) groups. Interestingly, in the ω-3+ω-6-treated group, the DGLA levels showed an increase (p<0.001) on D90 (1.21±0.05) compared with D0 (1.05±0.04) but did not show a significant difference on D30. By contrast, on D30 of treatment, DGLA levels were lower (p<0.0001) in the ω-3-treated group (0.89±0.01) than on D0 (0.96±0.01). At the end of the study, on D90, there were no fluctuations in the levels of AA, EPA and DHA, and the AA/EPA ratio remained approximately constant in all groups compared with D30.

**Table 2:** Fatty acid analysis from blood samples collected on D0, D30 and D90 from groups **A**, C57BL/6 untreated; **B**, CCL2^−/−^ untreated; **C**, CCL2^−/−^ treated with ω-3+ω-6 and **D**, CCL2^−/−^ treated with ω-3

	DGLA	AA	EPA	DHA	AA/EPA
**D0**					
**A**	1.05 ±0.04	7.04 ±0.10	1.59±0.04	3.24±0.09	4.48±0.13
**B**	1.23±0.04	7.08±0.09	1.62±0.05	3.29±0.09	4.38±0.14
**C**	1.05±0.04	6.99±0.09	1.48±0.04	3.16±0.08	4.80±0.14
**D**	0.96±0.01	7.26±0.17	1.39±0.09	3.77±0.06	5.52±0.23
**D30**					
**A**	1.07±0.04	6.97±0.10	1.50±0.05	3.15±0.09	4.74±0.15
**B**	1.07±0.04	7.00±0.10	1.51±0.04	3.18±0.09	4.71±0.14
**C**	1.13±0.05	4.99±0.16	4.57±0.16	4.43±0.14	1.10±0.20
**D**	0.89±0.01	5.01±0.17	4.56±0.17	4.42±0.15	1.10±0.02
**D90**					
**A**	1.02±0.04	6.91±0.10	1.44±0.05	3.09±0.09	4.90±0.16
**B**	1.17±0.04	7.12±0.10	1.60±0.05	3.27±0.09	4.71±0.14
**C**	1.21±0.05	5.07±0.16	4.65±0.16	4.51±0.14	1.10±0.02
**D**	0.97±0.01	5.02±0.17	4.64±0.17	4.61±0.18	1.10±0.04

Gas chromatography was used to obtain the results.

Data are expressed as the mean±SEM (n=22–24).

Fatty acid analysis from blood samples collected on D0, D30 and D90 from groups **A**, C57BL/6 untreated animals; **B**, CCL2^−/−^ untreated; **C**, CCL2^−/−^ treated with ω-3+ω-6; **D**, CCL2^−/−^ treated with ω-3. GC was used to obtain the results. Data are expressed as the mean±SEM (n=22–24).

### Retinal fatty acid analysis

**Figure 2:**
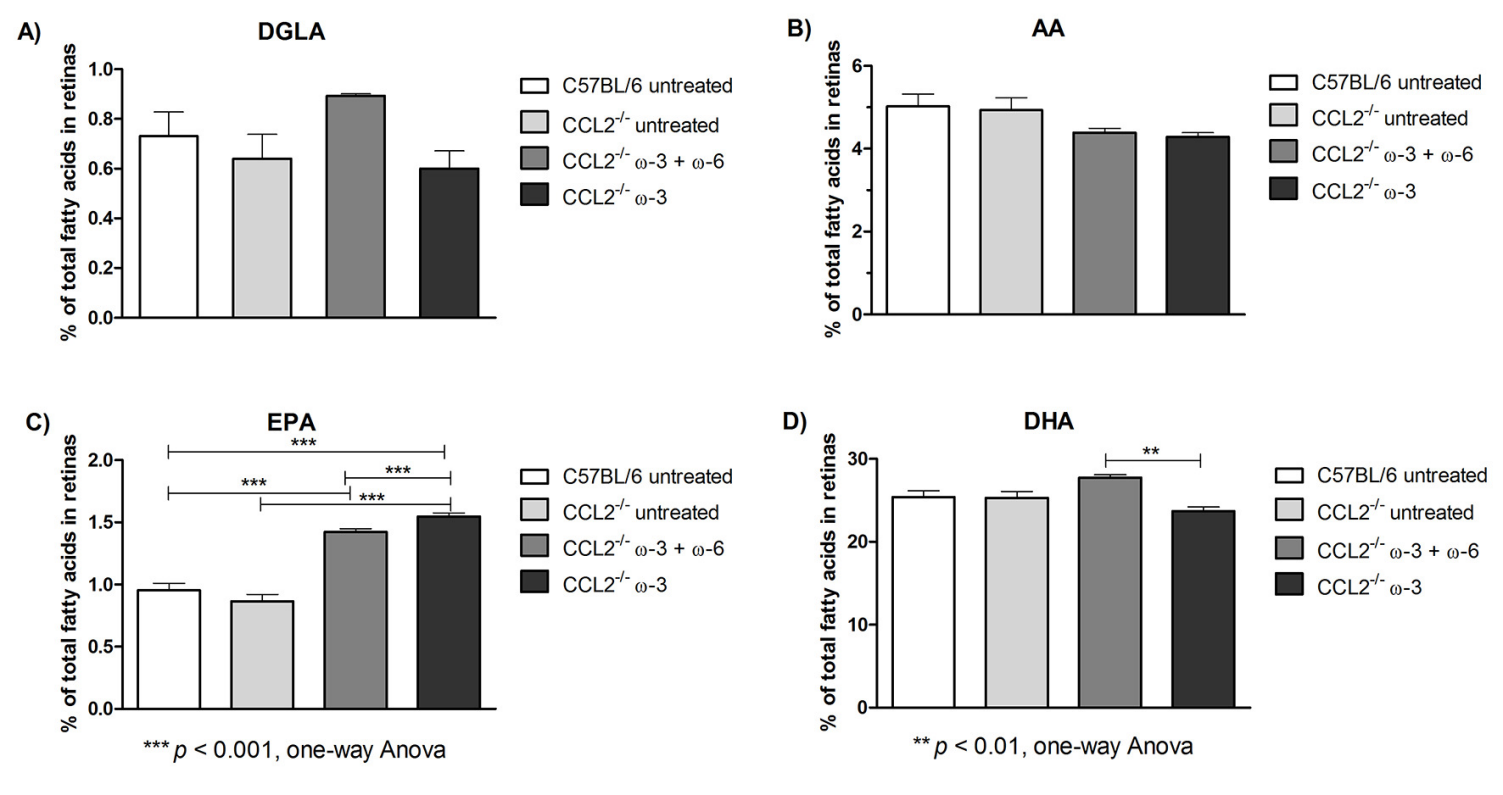
Fatty acid analysis from retina samples collected on D90 from groups A, C57BL/6 untreated; B, CCL2^−/−^ untreated; C, CCL2^−/−^ treated with ω-3+ω-6; and D, CCL2^−/−^ treated with ω-3. The proportion of DGLA, AA, EPA and DHA is expressed as the mean±SEM (n=4).

In addition, retinas from each group were collected on D90, and the percentage of retinal fatty acids, including DGLA, AA, EPA and DHA, was also determined using GC, as shown in figure 2 and table 3. The level of retinal DGLA in the ω-3+ω-6-treated group (0.89±0.01) was significantly higher (p<0.05) than that in the CCL2^−/−^ untreated group (0.64±0.10). The DGLA level in the ω-3-treated group (0.60±0.07) was lower (p<0.01) than that in the ω-3+ω-6-treated group. The AA content was significantly reduced in both ω-3 (4.29±0.10, p<0.05) and ω-3+ω-6 (4.39±0.1, p<0.01) treated groups (compared with the CCL2^−/−^ untreated group (4.93±0.30)). Interestingly, there was an increase in EPA retinal levels in the ω-3+ω-6-treated group (1.42±0.03, p<0.001) and in the ω-3-treated group (1.55±0.03, p<0.001), compared with the CCL2^−/−^ untreated (0.87±0.06) or wild type (0.99±0.05) groups. As expected, retinal DHA levels were the highest of all fatty acids, at approximately 25-fold. An increase (p<0.05) in DHA was observed in the ω-3+ω-6-treated group (27.73±0.36) compared with the CCL2^−/−^ untreated (25.30±0.77) and wild type (25.09±0.87) groups, whereas in the ω-3-treated group (23.70±0.51), there was a substantial reduction (p<0.001) compared with the ω-3+ω-6-treated group.

**Table 3:** Fatty acid analysis from retina samples collected on D90 from groups A, C57BL/6 untreated animals; B, CCL2^−/−^ untreated; C, CCL2^−/−^ treated with ω-3+ω-6; D, CCL2^−/−^ treated with ω-3

	DGLA	AA	EPA	DHA
**D90**				
**A**	0.65 ± 0.06	5.11 ± 0.35	0.99 ± 0.05	25.09 ± 0.87
**B**	0.64 ± 0.10	4.93 ± 0.30	0.87 ± 0.06	25.30 ± 0.77
**C**	0.89 ± 0.01	4.39 ± 0.11	1.42 ± 0.03	27.73 ± 0.36
**D**	0.60 ± 0.07	4.29 ± 0.10	1.55 ± 0.03	23.70 ± 0.51

Gas chromatography was used to obtain the results.

Data are expressed as the mean±SEM (n=4).

### Immunofluorescence staining

**Figure 3:**
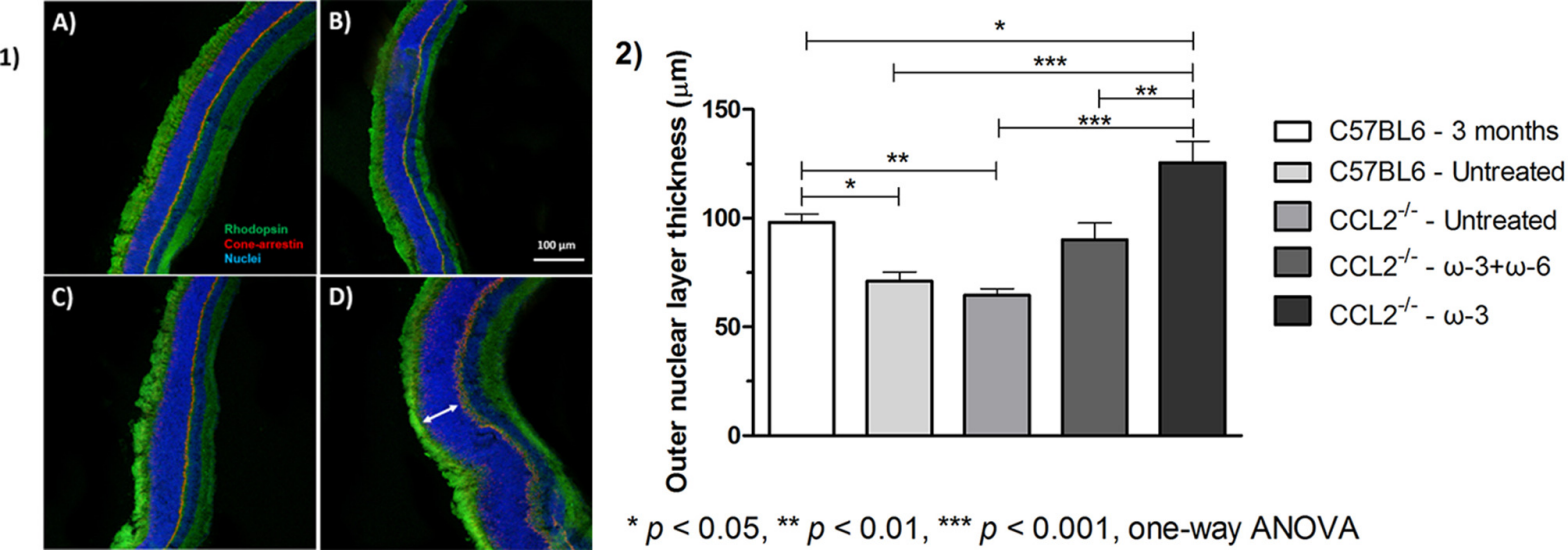
(1) Representative ocular photomicrographs of groups A, C57BL/6 untreated; B, CCL2^−/−^ untreated; C, CCL2^−/−^ treated with ω-3+ω-6; and D, CCL2^−/−^ treated with ω-3. Cryosections were stained for cone arrestin (red), rhodopsin (green) and TO-PRO-3 iodide (blue) for nuclear staining. Samples were examined by a Leica TCS SP5 confocal microscope (original magnification 100×, scale bar=50 µm). Arrow indicates retinal ONL. (2) Measurements of the ONL were performed at different fields around the entire retinal section (centre, middle and periphery). ImageJ software was used to calculate the ONL thickness, and the mean ONL thickness of the entire retina was compared among the different groups. Data represent the mean±SEM (n=5).

The ONL thickness was examined in control and treated groups, as presented in figure 3. No difference in the ONL thickness was observed between the aged wild type (12 months old) and the aged-matched CCL2^−/−^ animals (71.1±4.2 µm and 65.6±3.0 µm, respectively). Previous studies have reported that the CCL2^−/−^ model demonstrates confluent areas of visible atrophy after 16 months of age^34^; therefore, no changes were expected to be seen at this point. By contrast, the younger C57BL/6 mice (3 months old) exhibited significantly (p<0.05) greater ONL thickness (98.0±3.9 µm) compared with both wild type and CCL2^−/−^ animals. Both treatment groups, ω-3+ω-6 and ω-3 only, exhibited significantly larger ONL thicknesses than the untreated CCL2^−/−^ animals, with a more pronounced effect in the ω-3-treated group (90.0±7.8 µm (p<0.05) and 125.6±9.8 µm (p<0.001), respectively, compared with 65.6±3.0 µm). Interestingly, the ONL thickness was even greater in the ω-3-treated group than in the young animals (125.6±9.8 µm compared with 98.0±3.9 µm, respectively, p<0.05). Quantification of rhodopsin and cone arrestin could not accurately be performed from the immunostaining; for that reason, the ONL thickness was the only measurement that was carried out in the histological analysis.

**Figure 4:**
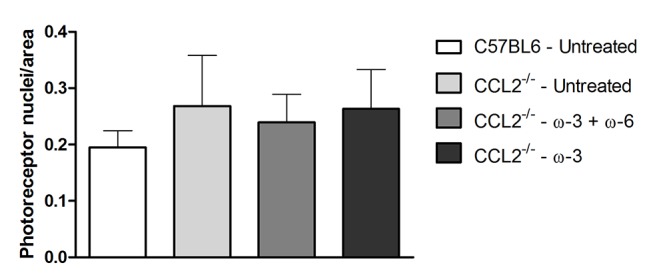
Photoreceptors’ density as estimated by ImageJ among the different groups (photoreceptors nuclei number/retinal area). Data represent the mean±SEM (n=3).

In order to exclude the possibility that the increase in ONL was due to nuclei swelling or spacing, the density of photoreceptors was calculated by dividing the number of nuclei over the retinal area (figure 4). There was no significant difference determined in any of the groups (0.19±0.03 in C57BL/6 untreated group, 0.27±0.09 in CCL2^−/−^ untreated group, 0.24±0.05 in ω-3+ω-6-treated group and 0.26±0.07 in ω-3-treated group).

### qRT-PCR analysis

**Figure 5:**
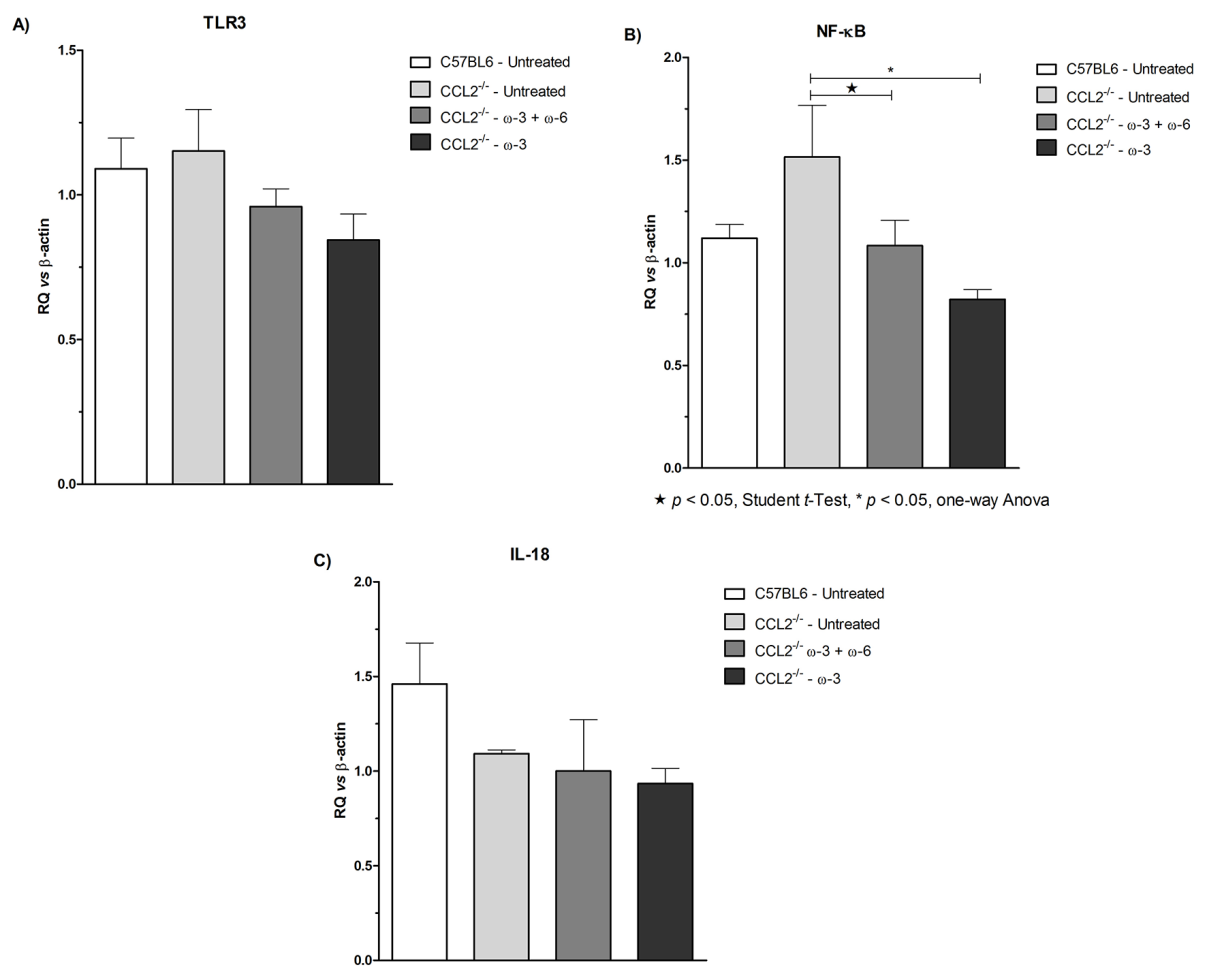
Gene transcripts of (A) *TLR3*, (B) *NF-κB* and (C) *IL-18* in the retinas of C57BL/6 untreated, CCL2^−/−^ untreated, CCL2^−/−^ treated with ω-3+ω-6 and CCL2^−/−^ treated with ω-3. Lower levels in *TLR3* and *NF-κB* mRNA were detected by quantitative reverse transcription PCR in the ocular tissue of both treatment groups. The graph is plotted as the mean±SEM (n=4–5).

The response of treatment with regard to the regulation of inflammatory mediators, in particular, the mRNA levels of *TLR3*, *NF-κB* and *IL-18* in retinal tissues, was examined using qRT-PCR analysis, as shown in figure 5. The expression levels of *TLR3* were lower, although not significantly, in both treatment groups than in the untreated group, with a more evident effect in the ω-3-treated group. A significant (p<0.05) reduction in the expression levels of *NF-κB* was observed in both treatment groups compared with the CCL2^−/−^ untreated group. However, there was no apparent difference in the expression levels of *IL-18* in either treatment groups and those in the CCL2^−/−^ untreated group, the only notable effect being an increased expression in the wild type animals.

### Western blot analysis

**Figure 6:**
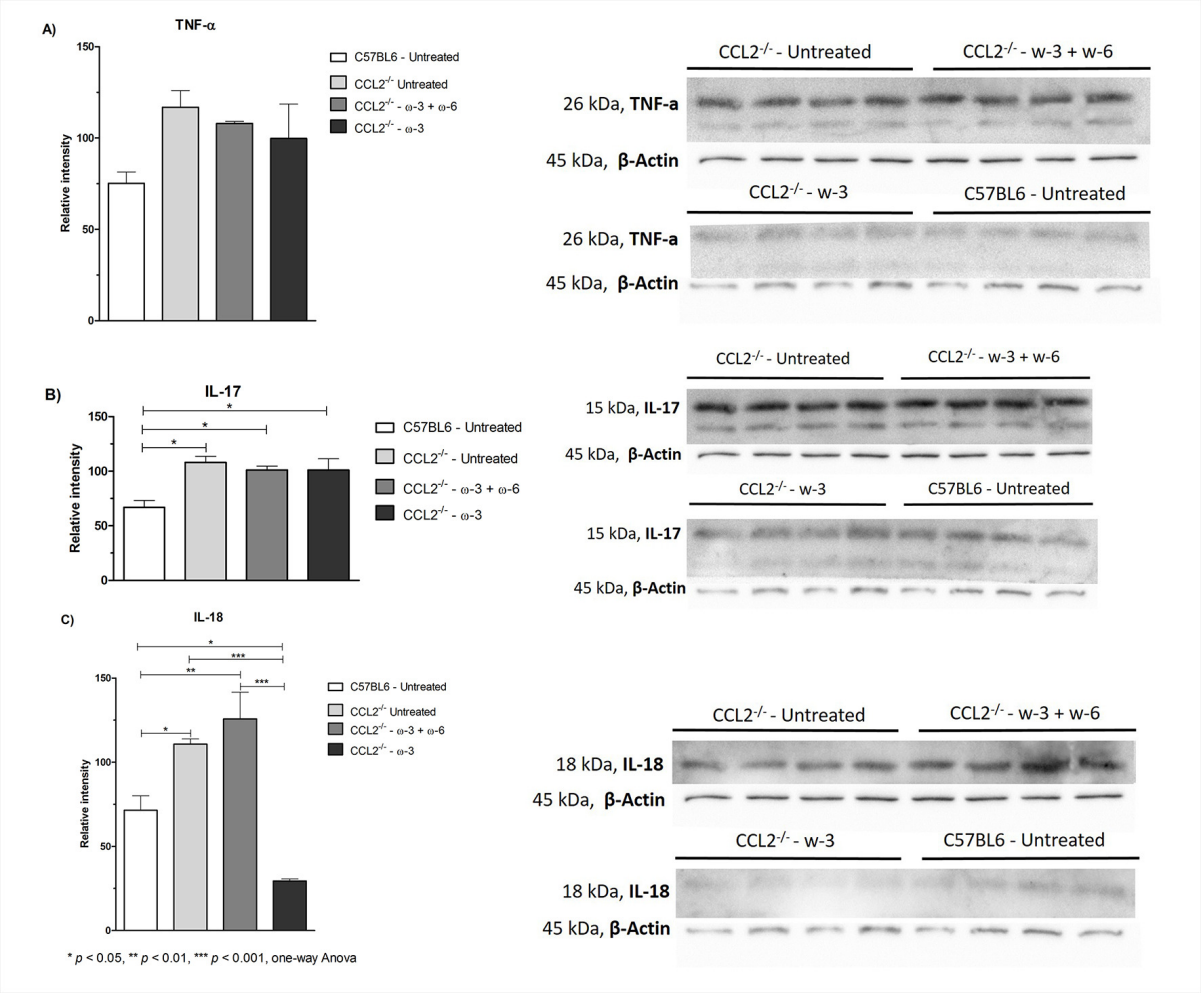
Western blot analysis of (A) TNF-α, (B) IL-17 and (C) IL-18 protein levels in the retinas of C57BL/6 untreated,  CCL2^−/−^ untreated, CCL2^−/−^ treated with ω-3+ω-6 and CCL2^−/−^ treated with ω-3. A significant reduction in the protein levels of IL-18 was observed in the ω-3 treatment group only. The graph is plotted as the mean±SEM (n=4-5).

To further investigate the effect of treatment on the inflammatory response, retinal tissues were evaluated to identify any difference in the protein levels of TNF-α, IL-18 and IL-17, as shown in figure 6. A minor reduction was observed in the TNF-α levels in both treatment groups compared with the untreated group; however, no effect was noted in the IL-17 levels. Interestingly, a significant (p<0.001) 4-fold reduction in the IL-18 levels was observed in the ω-3-treated group compared with the CCL2^−/−^ untreated group but not in the ω-3+ω-6-treated group. In addition, the IL-18 protein level in the ω-3-treated group was even lower than that in the wild type group (p<0.01).

## Discussion

AMD is one of the leading causes of blindness worldwide. Although it is a common condition, no treatment to date has provided evidence of disease regression. However, several studies have focused on the importance of para-inflammation in the pathogenesis of AMD^8^; therefore, aiming to stabilise the disease using anti-inflammatory approaches is considered a reasonable strategy. ω-3 PUFAs have been used in numerous studies, both in preclinical and clinical settings, demonstrating their effectiveness against heart disease, cancer, diabetes and neurological and autoimmune diseases.^16^ Moreover, emphasis has been given in the treatment of ocular pathologies using ω-3 PUFAs.

The aim of this study was to investigate the effect of either ω-3+ω-6 PUFAs or ω-3 only in a CCL2^−/−^ animal model that shares some of the clinical features of human AMD,^34^ while monitoring the AA/EPA ratio (~1–1.5). Supplementation with PUFAs served more as a preventive measure than as a therapeutic regime because the CCL2^−/−^ model does not demonstrate signs of photoreceptor loss before the age of 16 months.^34^ The study was terminated when the animals were 12 months of age; therefore, there was no significant difference in the retinal ONL of CCL2^−/−^ and the wild type group. However, a significant increase in the ONL thickness was observed in both treatment groups, with a more pronounced effect in the ω-3-treated group.

A recent study by Georgiou *et al* demonstrated the neuroprotective effects of ω-3 in a rat model of anterior ischaemic optic neuropathy, through different actions, that is, preventing apoptosis of retinal ganglion cells, decreasing inflammatory cell infiltration and regulating macrophage polarisation to decrease the cytokine-induced injury of the optic nerve.^35^

Other studies, in particular, Tuo *et al*, used the double-knockout Ccl2^−/−^/Cx3cr1^−/−^ mice to investigate the effect of a high ω-3 PUFA diet, which included 1.9 wt % of each EPA and DHA, 0.66 wt % of α-linolenic acid and 0.4 wt % of docosapentaenoic acid. The ω-6/ω-3 ratio was 2.9, and the ω-6 source was linoleic acid only. Specifically, animals that ingested a high ω-3 diet up to 8 months of age showed progression of retinal lesions and minor photoreceptor loss relative to the low ω-3 diet group. This effect was suggested to occur through a reduction in the AA metabolism, as demonstrated by the decreased pro-inflammatory derivatives (PGE_2_ and LTB_4_).^22^ High levels of dietary ω-3 PUFAs may result in the incorporation of EPA into cell membrane phospholipids at the expense of AA, leading to less substrate being available for eicosanoid synthesis.^36^

Furthermore, Ramkumar *et al* reported the effect of the AREDS2 formulation in the same Ccl2^−/−^/Cx3cr1^−/−^ model.^20^ This formulation included ω-3 fatty acids (54.9 mmol EPA/kg diet and 25.2 mmol DHA/kg diet), 17.6 mmol lutein/kg diet and 1.76 mmol zeaxanthin/kg diet. After 3 months of treatment, histological analysis demonstrated that the ONL thickness was greater in the high ω-3-treated group than that in the Ccl2^−/−^/Cx3cr1^−/−^ control-diet group. Additional studies reported the effect of ω-3 supplementation on the ONL in a light-induced retinal degeneration model, whereby animals treated with fish oil and anti-oxidants preserved their retinal structure and function.^37^

Our findings indicated a superior effect with regard to photoreceptor preservation because the ω-3-treated group exhibited an even greater ONL thickness than that of younger mice (3 months old). There was no difference in the number of photoreceptors per area when a comparison was performed between different groups, indicating that the increased ONL thickness was due to an increased number of cells and not due to any swelling effect. However, additional studies need to investigate this phenomenon, using 5-bromo-2-deoxyuridine to test for the existence and nature of any proliferating cells.

It has been hypothesised that the positive effects of ω-3 PUFAs (eg, EPA and DHA) is due to the formation of their metabolites, which possess anti-inflammatory properties. The production of pro-inflammatory eicosanoids is generated from the ω-6 PUFAs (eg, AA and GLA), which can act as mediators for leucocyte chemotaxis and inflammatory cytokine production. The balance between the pro-inflammatory and anti-inflammatory molecules is thought to be of great significance in disease progression. Therefore, in this study, we investigated the levels of fatty acids in blood and retinal tissues; in particular, the levels of DGLA, AA, EPA and DHA were monitored during treatment. The findings showed reduced pro-inflammatory and increased anti-inflammatory fatty acids in both blood and retina in the treatment groups. DHA is abundantly expressed in the photoreceptors, and vital retinal functions depend on its existence^38^; this influence was confirmed by its high retinal levels compared with the rest of the fatty acids. By contrast, EPA levels, although they were higher in the treated groups, were approximately 25-fold lower than those of DHA. This observation is explained by EPA’s greater metabolism by β-oxidation in the brain, which implies a shorter half-life, and by its low level of recycling into brain phospholipids.^39^ The gene expression and protein levels of some pro-inflammatory mediators were examined to find a correlation between the levels of fatty acids and the stage of the inflammatory response. A decrease in the transcript levels of retinal *TLR3* and *NF-κB* was observed in the treatment groups, with a more evident effect in the ω-3 group. TLR3 is associated with microglia cell activation, which is the brain's principal resident immune cell population, central to the inflammatory response and activated by distress signals released from neighbouring cells.^40^ On microglia activation, through TLR3, production of ILs^41^ and other cytokines is stimulated. Regulation of the transcription of inflammatory target genes (eg, *TNF-α*, *IL-6* and *IL-1*) can take place *via* NF-κB activation.^42^ Therefore, reduction of *TLR3* and *NF-κB* mRNA levels may indicate a reduced profile of microglia activation in the retina and subsequent decrease in the production of inflammatory cytokines. Microglia morphology was also examined in our study groups, but the differentiation between resting, activated and rod microglia was limited (data not shown).

No changes were observed in the gene expression of *IL-18*, in contrast to the protein levels, in which a significant decrease was observed in the ω-3 group, suggesting that a post-translational modification might have occurred, although further studies are required in order to establish this. Doyle *et al* reported that drusen isolated from donor AMD eyes activates the NLRP3 inflammasome, causing secretion of IL-18 and IL-1β.^43^ In addition, IL-18 was found to attenuate choroidal neovascularisation in a wet AMD model,^44^ which demonstrated its importance in disease progression. TNF-α protein levels showed a minor decrease in both treatment groups (with a more profound effect in the ω-3 group); however, IL-17 did not exhibit any changes, possibly due to its lack of direct involvement in the CCL2^−/−^ model.

Previous studies have demonstrated that, in the Ccl2^−/−^/Cx3cr1^−/−^ model, lower ocular *TNF-α* and *IL-6* transcript levels are observed in animals fed with high ω-3 diet, suggesting that reactive mediators of ω-3 PUFAs may regulate differential gene expression.^22^ Additional studies have confirmed this connection by demonstrating that the retinal expression of *TNF-α*, *Cox-2*, *IL-1β*, *VEGF* and *iNos* is much lower in a high ω-3 treated group than in a control.^20^ Connor *et al* evaluated the therapeutic effect of ω-3 PUFAs on hypoxia-induced pathological neovascularisation in a mouse model of oxygen-induced retinopathy. The results suggested that, by increasing the level of ω-3 PUFAs either by dietary or genetic means (using a Fat-1 transgenic model that converts ω-6 to ω-3 PUFAs), there was a reduced hypoxic stimulus for neovascularisation. This effect was mediated through the bioactive metabolites neuroprotectinD1, resolvinD1 and resolvinE1, through reduction in the *TNF-α* expression.^45^

It is evident from numerous preclinical studies and several clinical and observational studies that there is a clear association of ω-3 PUFAs and ocular pathologies, in particular, with AMD.^27 46^ It is important to note that dietary consumption of fish plays a key role in disease progression. A meta-analysis review examined the relationship of fish consumption and its relation with AMD. A linear association was found between the dose of fish consumption and the risk of AMD, indicating that the more fish consumption a person might have, the lower the risk for AMD.^47^ Our findings suggest that treatment with ω-3 PUFAs has an effect on the resolution of inflammation, but this effect is not the only determinant factor for disease regression. There was a greater response in the ω-3-treated group than in the ω-3+ω-6-treated group with regard to their anti-inflammatory action; however, both types of treatments were sufficient to increase the retinal ONL thickness. Although there is no direct explanation for the pronounced effect of ω-3 treatment with regard to the number of photoreceptors, it is hypothesised that the mode of action of ω-3 PUFAs may involve a stimulatory effect on endogenous retinal stem cells, such as the Muller glial cells, the ciliary pigment epithelial cells and the RPE cells.^48^ Further work is encouraged to establish a better understanding of this effect. Nonetheless, supplementation with ω-3 PUFAs has been demonstrated to be beneficial for dry AMD regression when AA/EPA=1–1.5 in this mouse model, and this could be considered a potential therapeutic regimen for patients with dry AMD.
