# Correction: Therapeutic potential of omega-3 fatty acids supplementation in a mouse model of dry macular degeneration

**DOI:** 10.1136/bmjophth-2016-000056corr1

**Published:** 2017-11-14

Prokopiou E, Kolovos P, Kalogerou M, *et al.* Therapeutic potential of omega-3 fatty acids supplementation in a mouse model of dry macular degeneration. *BMJ Open Ophthalmology* 2017;**2**:e000056. doi:10.1136/bmjophth-2016-000056.

There is an error in the Results paragraph of the Abstract. The sentence:

‘The outer nuclear layer thickness was increased in groups C (45.0±3.9 μm) and D (62.8±4.9 μm) compared with groups B (65.6±3.0 μm) and A (71.1±4.2 μm), and in young mice, it was 98.0±3.9 mm.’

Should have read,

‘The outer nuclear layer thickness was increased in groups C **(90.0±7.8 μm)** and D **(125.6±9.8 μm)** compared with groups B (65.6±3.0 μm) and A (71.1±4.2 μm), and in young mice, it was 98.0±3.9 μm.’

